# Atrial septal defect in a pediatric patient with Williams Syndrome: a rare presentation

**DOI:** 10.1093/jscr/rjac527

**Published:** 2022-11-28

**Authors:** Javier Grajeda, Amir N Mubarak, Javier Ardebol, Guillermo Grajeda

**Affiliations:** Medical Research, Universidad Francisco Marroquin, Guatemala, 01010, Guatemala; Medical Research, Universidad Francisco Marroquin, Guatemala, 01010, Guatemala; Medical Research, Universidad Francisco Marroquin, Guatemala, 01010, Guatemala; Departamento de Pediatria, UNICAR, Guatemala, 01010, Guatemala

**Keywords:** Williams Syndrome, congenital heart defect, atrial septal defect

## Abstract

Characterized by congenital heart defects (CHD) and elfin-like facies, Williams-Beuren syndrome (WS) is a multisystemic disorder that occurs approximately in 1 in 10 000 newborns [1]. WS is caused by a contiguous gene microdeletion of the Williams Beuren syndrome critical region (WBSCR) on chromosome 7q11.23, resulting in an abnormal elastin gene (ELN). There is a wide range of CHD in patients with WS, with supravalvular aortic stenosis (SAS) being the most common, and atypically the atrial septal defect (ASD) [2]. Few reports and reviews have linked the appearance of ASD to WS. Thus, data on the management of ASD secondary to WS is not well-documented. The following case report consists of the diagnosis and management of an ASD in a pediatric patient with WS.

## INTRODUCTION

Williams-Beuren Syndrome is a genetic condition affecting multiple body systems [2]. Cardiovascular defects are frequently observed, the most common defect being supravalvular aortic stenosis (SVAS), followed by peripheral pulmonary artery stenosis. Generally, defects arise from vascular involvement making structural cardiac abnormalities, such as ASD, unlikely presentations [2]. Due to the wide spectrum of possible cardiac manifestations, patients must undergo proper cardiovascular screening. The present case consists of the diagnosis and management of an atrial septal defect secondary to Williams Syndrome in a pediatric patient.

## CASE PRESENTATION

**Figure 1:**
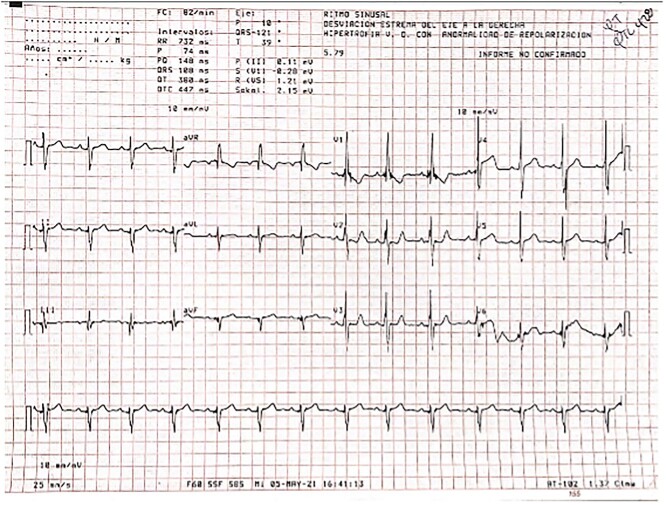
ECG displaying a normal sinus rhythm with right axis deviation suggestive of right ventricular hypertrophy

An 8-year-old male and his mother were referred to the cardiovascular surgery unit with a history of atrial septal defect associated with Williams Syndrome diagnosed at 6 months of age. Physical examination was relevant for distinct elfin facies, a broad forehead, flat nasal bridge, periorbital fullness, long philtrum, a bulbous nasal tip, malar flattening, wide mouth, small misaligned and widely spaced teeth, small jaw, and enlarged earlobes on inspection. A systolic murmur on the left 2^nd^ intercostal space was heard during auscultation. To assess the atrial septal defect and the possibility of surgical intervention, the treating physician requested an echocardiogram, cardiac catheterization, and an electrocardiogram. The echocardiogram confirmed an ostium secundum atrial septal defect with a left-to-right shunt. Catheterization showed a normal heart contour with dilation of the right atrium. Finally, the electrocardiogram (ECG) revealed a normal sinus rhythm with right axis deviation and right ventricular hypertrophy (Fig. 1). Due to the results of the diagnostic tests, closure of the septal defect via percutaneous trans-catheterization was performed. After the procedure, the patient was moved into the pediatric care department, where he continued his recovery. Postoperative assessments showed an alert and active patient, with a rhythmic heart and normal vital signs. The patient and his mother were discharged, with a treatment plan of furosemide and ibuprofen, and a one-month postoperative appointment. At the one-month follow-up, the patient showed adequate clinical progress.

## DISCUSSION

A microdeletion on chromosome 7q11.23 is a cardinal feature in the pathogenesis of the Williams Syndrome (WS) phenotype [1]. Patients develop a multisystemic disorder characterized by hypercalcemia, distinctive elfin-like facies, connective tissue and growth abnormalities, and variable levels of mental retardation. An important feature of the syndrome is the presence of congenital heart defects (CHD). The inheritance pattern of the microdeletion is of autosomal dominant manner, and leads to the deletion of a critical region that encompasses the elastin gene (ELN) [3], which is needed for normal cardiovascular development [4]. CHD occurs in approximately 80% of patients with supravalvular aortic stenosis (SVAS) being the most common presentation [4], occurring in 55% of patients [2]. SVAS is classified as a ‘typical cardiac defect’ in patients with WS. Nonetheless, this patient was diagnosed with an atrial septal defect, an ‘atypical cardiac defect’ [4], which has an incidence of 3–6% [2] in patients with the disease.

Nevertheless, this microdeletion is also responsible for the connective tissue phenotype of the patient [1]. The complete discussion of the pathophysiology of WS is beyond the scope of this article. However, due to the fact that cardiovascular defects are the most common cause of death in patients with WS [5] and the unusual cardiac presentation in this boy, we believe these findings are worth exploring and reporting. The atrial septal defect presented by the patient was of ostium secundum nature, meaning there was an increased reabsorption of the septum primum at the atrium’s roof or the septum secundum did not occlude the ostium secundum during heart morphogenesis [6]. The interatrial communication and the high-pressure difference led to development of a left-to-right shunt, for which direction and magnitude are determined by the size of the septal defect and pressure gradient [7]. Right atrial dilation seen with catheterization most likely resulted from, chronic volume overload secondary to shunted blood. The increased volumes and myocardial wall stress in the right atrium and ventricle ultimately lead to hypertrophy [7]. It logically follows that right axis deviation would be expected to be seen on ECG (Fig 1).

The diagnosis of WS is established both clinically and genetically. Initially, this syndrome is suspected with a physical exam and established with Fluorescence in situ hybridization (FISH) [8]. After confirmation, patients warrant cardiovascular screening. Cardiovascular evaluation consists of an ECG and echocardiogram. The use of other diagnostic tools will depend on the age and the severity of the lesion [5].

Management of the cardiovascular presentation will highly depend on the lesion, so patients can be initially treated non-operatively. Additionally, Collins *et al.* [5] suggests that patients suffering from WS with a concomitant cardiovascular abnormality should be examined every 3 months during the first year of life, and annually thereafter. In contrast, patients with significant ASD are commonly treated surgically as failure to intervene often leads to right-side heart failure [7]. Since ASD in patients with WS is rare, there are currently no specific guidelines for the surgical management of this cardiac abnormality. Despite this, patients with ASD secondary to ostium secundum defects requiring closure are treated with percutaneous trans-catheterization [6]. This minimally invasive procedure has its own advantages including a low post-interventional risk (7.2%) compared to open surgery (24%) [6]. This is pertinent to patients with WS as periprocedural sudden cardiovascular collapse has been reported exhaustively [2].

Physicians considering cardiovascular intervention must take into account current reports to improve management. Collins *et al.* [2] offers a procedural risk stratification system for these patients based on different pathophysiological factors, and provides a preoperative hydration plan according to the same risk stratum. Post-surgical pharmacological management of WS patients with ASD is not currently reported. Patients that undergo closure of ASD via a percutaneous trans-catheter require antiplatelet therapy for 6 months [6]. Those presenting with right heart hypertrophy as a result of volume overload benefit from loop diuretics like furosemide. This medication decreases sodium and chloride reabsorption in the thick ascending loop of Henle and thus increases diuresis [9]. Finally, every patient undergoing closure of ASD via percutaneous trans-catheterization require monitoring and regular follow-ups to evaluate right heart function and structure [6].

## CONCLUSION

Atrial septal defect as a cardiovascular manifestation in patients with Williams-Beuren Syndrome is infrequent, as vascular manifestations contribute to most cases. ASD must be considered as part of the differential diagnosis when managing a patient with the multisystemic manifestations of patients with Williams Syndrome. Although the septal abnormality is very uncommon, treating clinicians ought to consider this possibility due to the wide range of cardiovascular defects in these patients. Physicians are advised to acknowledge this unusual cardiac manifestation in patients with WS, regardless of the usual vascular presentations. For this reason, screening with echocardiogram and ECG are mandatory in all patients.

## CONFLICT OF INTEREST STATEMENT

None declared.

## FUNDING

None.

## References

[1] Morris CA . Introduction: Williams Syndrome. Am J Med Genet C Semin Med Genet. 2010;154C:203–8. 10.1002/ajmg.c.30266 PMC2946897 20425781

[2] Thomas R , Ii C. Cardiovascular disease in Williams syndrome. 2018; Available from: www.co-pediatrics.com.

[3] Adam MP , Mirzaa GM, Pagon RA. Williams Syndrome Synonym: Williams-Beuren Syndrome. 1999, 1993–2022.

[4] Yuan S-M . Congenital heart defects in Williams syndrome. Turk J Pediatr 2017;59:225–32. 29376566 10.24953/turkjped.2017.03.001

[5] Collins RT . Cardiovascular disease in Williams syndrome. Circulation 2013;127:2125–34. 23716381 10.1161/CIRCULATIONAHA.112.000064

[6] Menillo AM , Lee LS, Pearson-Shaver AL. Atrial Septal Defect. Case Stud Pediatr Anesth 2021 [cited 2022 Aug 11];277–81. Available from: https://www.ncbi.nlm.nih.gov/books/NBK535440/.

[7] Le Gloan L , Legendre A, Iserin L, Ladouceur M. Pathophysiology and natural history of atrial septal defect. J Thorac Dis. 2018;10:S2854. 30305945 10.21037/jtd.2018.02.80 PMC6174151

[8] Pober BR . Williams–Beuren Syndrome. N Engl J Med 2010;362:239–52. 20089974 10.1056/NEJMra0903074

[9] Casu G , Merella P. Diuretic Therapy in Heart Failure – Current Approaches. Eur Cardiol Rev 2015;10:42. 10.15420/ecr.2015.10.01.42 PMC6159465 30310422

